# Insights from the trial innovation network’s initial consultation process

**DOI:** 10.1017/cts.2025.10084

**Published:** 2025-06-26

**Authors:** Paul A. Harris, Nan Kennedy, Consuelo H. Wilkins, Karen Lane, Gordon R. Bernard, Jonathan D. Casey, Daniel E. Ford, Salina P. Waddy, Ken L. Wiley, Terri L. Edwards, Nichol McBee, Dixie D. Thompson, Mary Stroud, Emily S. Serdoz, Sarah J. Nelson, Michelle Jones, Lindsay M. Eyzaguirre, Leslie R. Boone, Jessica Baird, Colleen E. Lawrence, Elizabeth Holthouse, Sarah K. Cook, Maeve Tischbein, Natalya Amrine, Tiffany Chen, Jodie Cohen, LaShondra Deyampert, Natalie A. Dilts, Delicia Burts, Amna Baig, Joseph G. Christodoulou, Mariela Rodriguez, Edgar R. Miller, James F. Casella, W. Andrew Mould, J. Michael Dean, Daniel K. Benjamin, Harry P. Selker, Marisha E. Palm, Lori Poole, Jeri S. Burr, Sara Hassani, Angeline Nanni, Meghan Hildreth, Daniel F. Hanley

**Affiliations:** 1 Vanderbilt Institute for Clinical and Translational Research, Nashville, TN, USA.; 2 Department of Biomedical Informatics, Vanderbilt University Medical Center, Nashville, TN, USA; 3 Department of Medicine, Vanderbilt University Medical Center, Nashville, TN, USA.; 4 Department of Internal Medicine, Meharry Medical College, Nashville, TN, USA.; 5 Johns Hopkins University School of Medicine, Baltimore, MD, USA; 6 Duke Clinical Research Institute, Durham, NC, USA; 7 Johns Hopkins Institute for Clinical and Translational Research, Baltimore, MD, USA; 8 National Center for Advancing Translational Sciences, Bethesda, MD, USA; 9 University of Utah Health, Salt Lake City, UT, USA; 10 Utah Clinical & Translational Science Institute, Salt Lake City, UT, USA; 11 Duke University School of Medicine, Durham, North Carolina, USA; 12 Institute for Clinical Research and Health Policy Studies, Tufts Medical Center, Boston, MA, USA; 13 Tufts Clinical and Translational Science Institute, Tufts University, Boston, MA, USA

**Keywords:** Multicenter studies, Trial Innovation Network, Trial Innovation Center, Recruitment Innovation Center, scientific consultation

## Abstract

Multicenter clinical trials are essential for evaluating interventions but often face significant challenges in study design, site coordination, participant recruitment, and regulatory compliance. To address these issues, the National Institutes of Health’s National Center for Advancing Translational Sciences established the Trial Innovation Network (TIN). The TIN offers a scientific consultation process, providing access to clinical trial and disease experts who provide input and recommendations throughout the trial’s duration, at no cost to investigators. This approach aims to improve trial design, accelerate implementation, foster interdisciplinary teamwork, and spur innovations that enhance multicenter trial quality and efficiency. The TIN leverages resources of the Clinical and Translational Science Awards (CTSA) program, complementing local capabilities at the investigator’s institution. The Initial Consultation process focuses on the study’s scientific premise, design, site development, recruitment and retention strategies, funding feasibility, and other support areas. As of 6/1/2024, the TIN has provided 431 Initial Consultations to increase efficiency and accelerate trial implementation by delivering customized support and tailored recommendations. Across a range of clinical trials, the TIN has developed standardized, streamlined, and adaptable processes. We describe these processes, provide operational metrics, and include a set of lessons learned for consideration by other trial support and innovation networks.

## Introduction

Clinical trials are essential for evaluating the safety and efficacy of new and existing treatments or interventions across appropriate and representative populations, ensuring findings are reproducible. Studies that generate evidence (e.g., early phase trials, management trials using drugs or devices, behavioral intervention trials, comparative effectiveness studies, decentralized trials) among specialized populations typically require multicenter recruitment and enrollment to attain a sample size sufficient to ensure generalizable results within a meaningful timeline. Multicenter trials can speed the pace of scientific discovery and translation, but face ongoing challenges to successful completion, such as complexities in study design, coordination, and data management across multiple sites; lengthy design-test-implement cycles; recruitment and retention of all populations, including those with limited representation in research; and divergent interpretations by local Institutional Review Boards [1,2].

**Figure 1.**
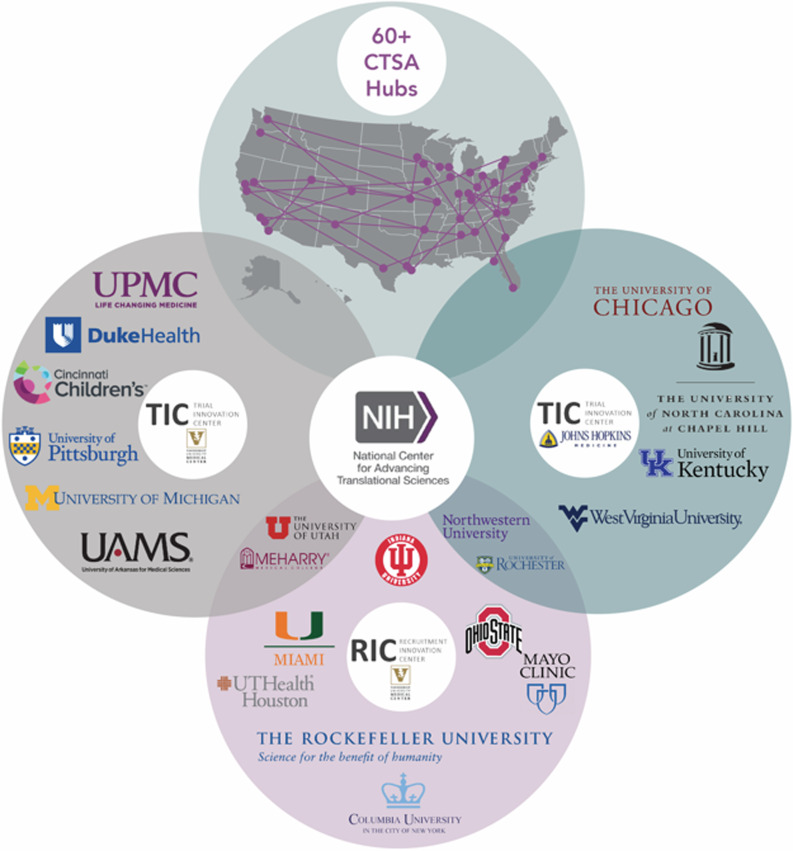
Trial Innovation Network partners, 2025. CTSA = Clinical and Translational Science Award.

Expert scientific and operational input is needed during trial planning stages to optimize designs, address methodological challenges, and ensure robust protocol development. The Trial Innovation Network (TIN) was established by the National Institutes of Health’s National Center for Advancing Translational Sciences (NCATS) as a collaborative initiative that seeks to accelerate the translation of research into clinical practice [3]. As part of this effort, experts and scientists from the Trial Innovation Centers (TICs), the Recruitment Innovation Center (RIC) [4], and Liaison Teams from >60 Clinical and Translational Science Awards (CTSA) Program Hubs across the country (Figure 1) collaborate in a multidisciplinary scientific consultation process to consider, plan, and conduct multicenter clinical trials while developing data-driven innovations and tools in trial design and operations.

Early network efforts and the original institutional configuration of innovation centers have been described by Bernard et al. [5], while the more mature network is discussed in recent publications [2,4,6–8]. The TIN’s freely available consultation process provides researchers access to a range of clinical trialists, methodologists, and disease domain experts, who offer feedback and guidance at various stages across the trial life cycle. By leveraging the multidisciplinary expertise of the TIN, and by extension the CTSA network writ large, investigators receive assistance in navigating the intricacies of study design and protocol development, multiple site coordination, regulatory variations, representative participant recruitment, quality data analysis, and broad dissemination of learned advances in science and medicine. Individual researchers, *including both new and seasoned principal investigators,* are empowered to optimize trial design and operational performance, understand and meet the needs of the patients at the core of their clinical question(s), and work together with TIN experts to accelerate the translation of promising scientific discoveries into real benefits for patients.

The TIN’s scientific consultations can include an Initial Consultation [9], targeted resources, customized recommendations, and/or a Comprehensive Consultation for investigators who wish to have one of the TICs serve as the data coordinating center or clinical coordinating center. This paper, which focuses on the Initial Consultation and targeted resources, describes the TIN’s current structure and areas of innovative emphasis, showcases examples of a variety of Initial Consultations, and presents a set of lessons learned that may help other trial network leaders design consultative services.

## Methods

### History of the TIN Initial Consultation

The TIN’s iterative proposal submission process began with an initial call for proposals in October 2016, yielding 38 proposals. In July 2017, we honed the submission process, committing to a 5-working-day response window during which a TIC or RIC manager would contact the study investigator to set up an introductory call and discuss expectations for the consultation process. Our revised process also ensured every investigator (100%) who applied to the TIN was offered an Initial Consultation with recommendations. Rolling acceptance, review, and consultative processes were developed to enhance program flexibility [2,5].

### Organizational structure

The guiding principle of the TIN Initial Consultation is to deliver early-stage, high-quality scientific consultations to investigators, tailored to address study-specific needs. To achieve this aim, the consultation process and governance structure have been standardized to enable rapid access to discussions among the principal investigator (PI) and study team, assigned TIC and RIC project leads, disease domain experts, CTSA members, and NIH program officers (Table 1), while also establishing connections with experienced TIN clinical trialists, biostatisticians, and recruitment experts. Consultative discussions may cover the study’s scientific premise, statistical design, recruitment and retention strategies, funding feasibility, and other support areas. In addition to fostering ongoing partnerships between the study team and consultation experts, the TIN encourages investigators to leverage the expertise and resources available within their own institutional CTSA program (when applicable) and promotes potential collaborations with external entities such as community hospitals and affiliated medical centers.

**Table 1.** Initial Consultation – TIN roles and responsibilities

Role	Responsibilities
TIC/RIC Principal Investigator (PI)	Contact PI or Co-PI for the Vanderbilt TIC, JHU TIC, and/or the RIC, who is responsible for the overall conduct of research studies within the TIC and RIC including thought leadership and compliance with applicable laws, regulations, and institutional policies governing the conduct of the sponsored research.
NIH Program Officer	Provides overall guidance and shares scientific and programmatic goals.
Domain Expert	Subject matter expert in a particular field, who is selected by each TIC and RIC to provide expert input and advice for Initial Consultations and requests for resources. Domain Experts play a key role in shaping final recommendations and products.
Biostatistician	Provides input on study design options and support for protocol development, statistical analysis plans, and sample size calculations. Their collaborative effort on consultations includes statistical consultation and access to advanced statistical tools. Biostatisticians are responsible for study design, statistical review, and interim and final analyses for all TIN studies.
Resource Lead	Responsible for assessing the study needs for each TIC/RIC resource and then ensuring these are delivered with high quality and consistency. Resource leads are identified for each of the TIC and RIC innovations and resources that are offered to study teams (e.g., sIRB, Community Engagement Studios, Expression of Interest, Site Assessment Survey Instrument, Clinician Study Application, ResearchMatch, Standard Agreements).
Consultation Lead	Responsible for reviewing study materials and working with PIs and study teams to provide evidence-based recommendations, advice, and guidance for Initial Consultations that are approved for TIC/RIC support.
Project Lead (PL)	Responsible for working closely with study teams, TIC/RIC PIs, Domain Experts, and Resource Leads to coordinate a varied range of consultations and resources for specific studies. They are the main point of contact for PIs and their respective study teams and ensure regular communication on progress and issues as they arise. The PL manages all proposals through the consultation and implementation processes and participates in TIN committees, working groups, and subgroups, and documents all data points to communicate progress through the TIN pipelines.
Local CTSA Hub Liaison Team (HLT)	Functions as an interface between the hub and the national collaborative activities of the CTSA Program, including activities of the TIN. The TIN has established infrastructure, communication flow, and processes to embed the local HLT into Network operations for representation and collaboration on individual trial opportunities and innovations. The HLT Medical Director and Point of Contact (POC) partner with the TICs and RIC for connecting local Investigators and trials with consultations and expert resources of the TIN, including participation in the consultation process to ensure local CTSA resources are optimized.
Proposal Assessment Team (PAT)	Meets on a biweekly basis to review proposals submitted to the TIN for a consultation. The broader teams provide input to the assigned TIC or RIC regarding the proposal and suggested support. The PAT voting members include two representatives from each TIC/RIC [6 voting members] and the Committee Chair [1 tie-breaking voting member], making 7 total voting members. Each individual TIC/RIC appoints its own voting members from its group of TIC/RIC investigators and project leads. Voting usually determines whether the TIN will enter into a Comprehensive Consultation with the investigator and which TIC will be assigned to the project.

TIN = Trial Innovation Network; TIC = Trial Innovation Center; RIC = Recruitment Innovation Center; JHU = Johns Hopkins University; NIH = National Institutes of Health; sIRB = single Institutional Review Board; CTSA = Clinical and Translational Science Award.

The TIN prioritizes proposals that can potentially test an operational or design innovation to enhance efficiency or reduce clinical trial costs. Initial Consultations are a first step; through them, the TIN discovers shared interest in partnering on methods to improve performance. From the needs and experiences communicated during collaboration with research teams across a multitude of studies and domains, the TIN identifies gaps in design and implementation, integrates new insights, and generates new resources, methods, and tools [6,10]. Using this bidirectional approach, the TIN has developed innovations such as the ResearchMatch Expert Advice Tool [11]; the IRB Reliance Exchange (IREx) to operationalize single IRB review and communications [12]; a module in REDCap to facilitate real-time participant randomization in adaptive trials; a site assessment survey instrument (SASI) [13]; and an accelerated trial start-up (ASU) program, among others [14].

### The TIN Initial Consultation process

A proposal to request a TIN consultation can be completed through the TIN website (trialinnovationnetwork.org) by any US-based investigator who is developing or conducting a multicenter study in any discipline and with any type of funding. The proposal captures the basic study design, objectives, endpoints, target population, intervention, study and participation duration, sites, requested resources (Table 2), funding source, and funding mechanism. If investigators are affiliated with a CTSA Hub, an acknowledgment or letter of support from their CTSA PI is required to ensure locally available resources (e.g., biostatistical support, study coordination support, and regulatory expertise) are leveraged when available. Following the review of the proposal, the TIN initiates and carries out the Initial Consultation. This process involves a series of consultative calls with the study investigator, domain experts, TIC or RIC lead, and relevant resource leads. Potential TIC/RIC resources (Table 2) that may be provided to the investigator are discussed, and those that are deemed appropriate and beneficial are implemented. The TIN provides a final Recommendations Report for all Initial Consultations, and if warranted by the needs of the study and approved by the Proposal Assessment Team (PAT), a TIN Comprehensive Consultation may follow.

**Table 2.** Initial Consultation areas of innovative emphasis

Area of support	Description
Study Planning (Design, Budget, Timelines, Feasibility)	*Design* A review and discussion of the submitted study proposal may cover the study goals and aims, design and methodology, statistical and regulatory considerations, participant recruitment, schedule of assessments, study interventions, or other components of the study, with the goal of working through potential barriers to successful study completion. *Budget* The TIN can evaluate the overall study budget, site and participant budget, and recruitment budget and then provide recommendations and estimates for proposed alterations based on the results of the Initial Consultation [10 ]. *Timelines* Timelines for submission of the application can be evaluated as well as timelines for the overall progression of the study, including planning, study start-up, conduct, close-out, and final publication. *Feasibility* The TIN can assess study feasibility and provide recommendations to optimize its successful completion within the proposed timelines and budget.
Innovative Trial Design	Trial design is addressed in every consultation. Our experts explore how to innovatively address trial complexities and gaps from bench to bedside. The TIN’s unique process of innovating trial designs accelerates the translation of novel interventions into life-saving therapies. Individualized, study-specific consultations Biostatistical, operations, and clinical experts jointly design strategies Innovative multicenter study designs, for example, adaptive, platform, pragmatic, and remote trials Methodologies may include Bayesian methods, master protocols, surrogate outcomes, and others Novel and proven TIN resources to accelerate trial operations, contracting, site selection, sIRB, and coordination Decentralized and/or hybrid trial design may improve research outcomes and representative recruitment, leveraging such tools as MyCap to capture remote patient-reported outcomes [15]. Identifying the role of the Data Safety Monitoring Board (DSMB) [16] can enhance the integrity and safety of clinical trials.
Single IRB (sIRB)	For trials requesting sIRB, the TIN can provide resources, coordination support, tools, and access to a web-based platform (IREx) [12] to operationalize the sIRB [17]. The cost for these resources is discussed during the TIN Initial Consultation.
Standard Agreements	The TIN can provide a Standard Agreement (modeled after the FDP-CTSA agreement) for use by each of the participating institutions in the investigator’s multicenter study [14].
Expression of Interest: Site Identification	An Expression of Interest (EOI) is a formal announcement from the TIN that provides CTSA sites, their affiliates, and partners with opportunities to participate in potential trials, with the TIN serving as an intermediary between the study PI and the CTSA site. To implement the EOI, the TIN works with the study team to complete a competing trials assessment; to develop a formal outreach that may require sites to determine feasibility of study protocol or site budget or both; to conduct an EHR-based Cohort Assessment for assessment of available recruitment populations; and to identify a local site investigator.
Recruitment and Retention Plan [8]	An effective recruitment and retention plan brings together strategies for engaging and incentivizing specific population(s) of relevance for a trial. Key features include: providing recruitment advice and recommendations; assessing the likelihood of meeting predefined recruitment and retention goals; and offering tailored advice and recommendations on appropriate recruitment strategies to engage participants from a particular community.
Recruitment Feasibility Assessment	The recruitment feasibility assessment resource involves evaluating the potential success of conducting a clinical trial in a particular geographical region, with the overall objective of optimal project completion in terms of desired study population, timelines, targets, and cost.
Recruitment and Retention Materials	Recruitment and retention materials may include custom-written or verbal communication delivered through a range of multimedia channels and platforms to increase enrollment and retention. Key features of this TIN resource are reviewing recruitment material needs, including any dissemination plans; providing advice, recommendations, and templates to augment recruitment of participants for specific studies; ensuring recruitment of representative populations [18,19]; and sharing best practices.
Community Engagement Studio	A Community Engagement Studio [20] is a structured method of engagement that facilitates meaningful involvement of heterogeneous groups of stakeholders. Engagement studios can be used to obtain project-specific input. Key features of this resource include assessing the areas in which a studio has the potential to add the most value to a study; providing advice and guidance to investigators on when and how to conduct studios; and supplying recommendations on how a studio can be used to develop and refine study messaging and materials.
Electronic Health Record (EHR)-Based Tools and Resources	The TIN can help investigators consider ways in which their EHR system and data may be leveraged to optimize recruitment, improve study design, and inform site selection [21,22]. This resource includes Clinical Systems Optimization, in which the RIC works with clinical study teams and their recruitment sites to better understand their site-specific recruitment workflows, IT capabilities, and study protocol, with the goal of generating recommendations to enhance recruitment efforts through the EHR. Through EHR-based Cohort Assessment, the RIC provides expert clinical and technical review of the study’s recruitment population, high-level assessment, and potential creation of computable phenotyping.

TIN = Trial Innovation Network; sIRB = single Institutional Review Board; IREx = Institutional Review Board Reliance Exchange; FDP-CTSA = Federal Demonstration Partnership - Clinical Trials Subaward Agreement; CTSA = Clinical and Translational Science Award; PI = Principal Investigator; EHR = Electronic Health Record.

## Results

### Consultation reach

**Figure 2.**
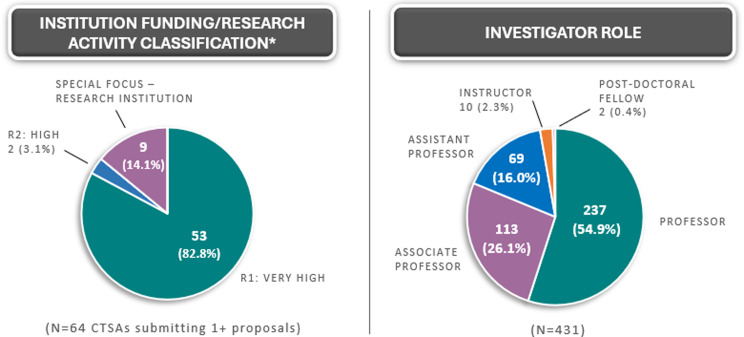
TIN Initial Consultations metrics – distribution of institution funding/research activity classification and investigator roles. R1= research 1; R2 = research 2. *Based on the Carnegie Classification of Institutions of Higher Learning©.

The TIN received 448 submissions requesting an Initial Consultation from October 2016 through June 1, 2024 (2016–44; 2017–42; 2018–78; 2019–67; 2020–78; 2021–46; 2022–41; 2023–34; 2024–20). Some proposals did not move forward or are in progress (*n* = 4) or were part of the TIN pilot/demonstration project (*n* = 13). In total, 431 TIN Initial Consultations were completed. Investigators receiving Initial Consultations are often from well-funded R1 institutions, as CTSAs are typically housed in large research centers (Figure 2). Nearly 20% of TIN Initial Consultations were delivered to junior investigators, including fellows, instructors, and assistant professors.

### Selected examples of Initial Consultations and outcomes

The following use cases present a range of studies with a variety of challenges, resources received, and outcomes that illustrate the possible progression of an Initial Consultation and its impact on a clinical trial.

**BEACH (Biomarker and Edema Attenuation in IntraCerebral Hemorrhage) Trial: Johns Hopkins University (JHU), University of Kentucky and JHU TIC, and Vanderbilt University Medical Center (VUMC) RIC.** Proposal for first-in-human evaluation of an anti-neuroinflammatory, small-molecule drug candidate in an adult acute trauma population, informed by evidentiary preclinical data. Initial Consultation design experts and content-specific physician scientists recommended shifting the focused population to one that is easier to assess, where factors influencing disease development are known and quantifiable. The TIN leveraged the CTSA program for EHR-based cohort assessments to identify optimal sites, assisted with establishing a Clinical Coordinating Center and Data Coordinating Center collaboration, and identified potential trial implementation innovations including automating near real-time brain CT scan measurement in screening and endpoint assessment. **Consultation Outcome: Design strategies, site identification, collaborative structure. A subsequent TIN Comprehensive Consultation resulted in a successful collaborative grant proposal to the National Institute of Aging, which was funded on the first attempt. NCT05020535**

**Physical Activity in People with Multiple Sclerosis (MS) Study: Columbia University and VUMC TIC, VUMC RIC.** Proposal for a Phase II, randomized, placebo-controlled trial in an adult autoimmune disease population, to evaluate medication dosing and physical activity levels. Initial Consultation design experts suggested expanding the intervention and designing a three-arm trial with placebo and recommended continuously monitoring activity levels rather than pre- and post-intervention. The ResearchMatch [23] Expert Advice Tool [11] collected pre-grant opinions from volunteers with appropriate lived experience. 61 volunteers responded, 72% said they would be “likely” or “extremely likely” to participate in the trial with continuous activity monitoring. The TIC recommended additional resources once funded: MyCap [15] for remote capture of patient-reported outcomes and the Clinician Study App [4] to facilitate provider referrals. Unsupportable budget impacts arose two weeks prior to grant submission, and were immediately addressed and mitigated by the VUMC TIC. The study PI was connected to an expert pharmacy resource; a revised quote was provided within 1 business day at <25% of the original pharmacy costs ($1M+ vs. $172K). **Consultation Outcome: Design strategies. Recommendations Report and Resources. Ongoing team collaborations. Grant not yet funded.**

**REBIRTH (Randomized Evaluation of Bromocriptine in Myocardial Recovery Therapy for Peripartum Cardiomyopathy) Study: University of Pittsburgh and VUMC RIC, Duke University TIC**. Proposal to evaluate the use of bromocriptine for myocardial recovery therapy in women living with peripartum cardiomyopathy at 50 recruitment sites. Anticipated challenges included: delayed care due to a lack of awareness of peripartum cardiomyopathy among providers; later diagnosis and care for Black women compared to white women; the need for genetic sample collection; and loss to follow-up. Hospitalization at enrollment was expected to be problematic if individuals were too sick or needed complex care that made them ineligible to participate. To support the grant submission, the RIC conducted a Community Engagement Studio [16] with priority populations to illuminate potential barriers and enable creation of a Recruitment and Retention Plan to mitigate risks. The RIC also developed an electronic phenotype algorithm to identify sites with the relevant rare disease population. **Consultation Outcome: Recommendations Report and Resources, Letter of Support for grant submission. Grant was funded. NCT05180773**

**WIRED UP Study: Johns Hopkins University and JHU TIC, VUMC RIC**. Open-label randomized controlled trial to determine if specialized insoles with real-time feedback and remote monitoring can prevent recurrent diabetic foot ulcers in adults with recently healed ulcers. Also assessed were patient satisfaction, quality of life, and healthcare costs compared to standard non-interactive insoles. The investigator initially tested the device at a single site, but wanted to expand to a multicenter study. After an intensive learning experience on the many requirements (such as sIRB), investigator and site selection, length of time to conduct a multicenter trial, and most importantly, cost of conducting a multicentered trial, the investigator concluded her budget could not stretch to multiple sites without compromising quality, and decided instead to focus on strengthening single site data collection. **Consultation Outcome: Recommendations Report. Investigator highly satisfied with the training and recommendations provided; plans to return to the TIN when transitioning to a multicenter trial.**

**Maternal and Infant Outcomes Study: Maternal and Infant Outcomes among Incarcerated Women Who Give Birth in Custody: University of Minnesota Twin Cities and University of Utah TIC.** Proposal to conduct a multicenter study of perinatal support for incarcerated women in five geographically representative prisons. The study aims to provide actionable insights for prisons when implementing perinatal support and doula programs to improve maternal and infant health outcomes. With funding secured, the Utah TIC collaborated to execute a single IRB (sIRB). Each participating state prison required partnerships with an academic institution and/or a community-based nonprofit organization. This funded study enables the Utah sIRB to facilitate meaningful stakeholder engagement and address challenges in conducting research with pregnant, incarcerated women. The Utah team worked with the study team to secure sIRB approval, establish reliance partnerships between the nonprofits, and navigate the intricacies of the Department of Corrections review and approval processes in each state. **Consultation Outcome: Approval for sIRB Support. Ongoing team collaborations.**

## Discussion

The low rate of successful completion of clinical trials in the United States constitutes an ongoing national research crisis. Recruitment and retention levels are suffering, costs continue to rise, and design issues produce uninformative data. These factors significantly curtail treatment advancement.

The Covid pandemic produced a dramatic negative impact on the viability of clinical trials for several years beginning in 2020, causing a reduction in the number of proposals for TIN Initial Consultations during that time period. The pandemic faded just as our TIN funding period began winding down. The uncertainty of award renewal for the RIC and TICs prevented us from aggressively promoting our services and likely caused investigators to hesitate in seeking out TIN resources. Since our funding was renewed in 2024, however, we have reinvigorated the dissemination and marketing of the TIN’s services. The number of TIN proposals received rose from 34 in 2023 to 55 in 2024, and we are on target to receive more than 55 in 2025.

The TIN provides scalable solutions to remediate these problems by providing timely advice and resources during initial clinical trial planning stages, at no cost to investigators. Its impact on clinical care is emerging: a recent analysis found that published articles from research supported by CTSAs were significantly more likely to be cited in health policy documents and clinical guidelines than the proportion expected [24]. The TIN contributes to this impact by accelerating clinical research so that research results can be more quickly translated into clinical practice.

As previously reported, investigators report high satisfaction with the consultation process and deliverables [9]. The TIN’s guiding principle is delivery of high-quality consultations that add value to an investigator’s study via a standardized, yet adaptable process tailored to the needs of each research team. For each Initial Consultation, the TIN leverages CTSA program expertise that complements local institutional resources.

### Lessons learned

Through experience, the TIN has learned that:

- A successful consultation starts with a strong scientific hypothesis, preferably backed by preliminary, supporting data.
- The TIN strongly recommends submitting a request for an Initial Consultation early in the grant proposal process (60–180 days before submission) to maximize consultation impact. Our consultations include experienced trialists who can often recommend funding sources or suggest strategies for funding submissions. Moreover, they can assist with proposal development, budget planning and resource allocation, and recruitment and retention planning to ensure that the trial is well-designed and appropriately resourced. Studies in need of “rescue” (i.e., studies already funded and in implementation needing additional assistance) often struggle to recruit individuals from populations with limited representation [25].
- Investigators may be approaching the TIN late in the process because of a lack of awareness. We have been expanding our marketing efforts through conference presentations, webinars, manuscripts, and word of mouth to inform investigators of the expertise freely available to them through a TIN consultation.
- Ideally, most sites should be US-based to foster broad collaboration within the CTSA Program and NIH Institutes and Centers.
- Consultations can prove especially beneficial for new investigators, newly organized consortia, or experienced investigators moving into new areas.
- Successful funding and study completion are not the only measures of a TIN Initial Consultation’s value. In some cases, a proposed study’s design is underdeveloped or under-resourced for a multisite trial. In those cases, the TIN may recommend that a study not move forward, and in doing so, we are often able to educate investigators on the many aspects of the multisite clinical trial process (sIRB, budgeting, recruitment and retention, site selection, etc.), save the investigator time and energy, and reduce the burden of the grants review process, placing them in a position to create a better proposal later with greater chance for success.
- Investigators at CTSA organizations should consult their local CTSA TIN Liaison to ensure a pragmatic consultation focus.

### Limitations

Although potentially open to any trialist, TIN Initial Consultation requests have primarily come from CTSA institutions. For studies not advancing to a Comprehensive Consultation, the TIN has limited ability to track outcomes (e.g., implementation of recommendations provided during consultations) outside of its consultative engagement with study teams. In addition, we are not aware of similar disease-neutral consultation networks outside the TIN to which its process or results can be compared.

### Future directions

The TIN will continue to develop new resources through iterative innovation discovery and development. We will continue to work to expand recruitment and retention to improve the representation of different patient groups, enhancing the likelihood of generating actionable and relevant results that benefit all patients. TIN’s Initial Consultation process awareness is being increased through seminars, conferences, and publications to attract new investigators and consortia. The TIN will continually refine its evaluation process to measure the impact of Initial Consultations and the effectiveness of tools and methods.

## Conclusion

The TIN’s well-developed infrastructure and wealth of resources help investigators plan, enhance, and expedite their multicenter clinical trials, thereby advancing new healthcare treatments for various diseases more rapidly. This model has been validated through numerous successful Initial Consultations, each tailored to meet an investigator’s specific needs and to overcome identified obstacles. TIN leadership continuously identifies recurring issues that hinder the timely completion of clinical trials and develops new resources to mitigate them. These innovations, incorporated into the TIN’s toolbox of offerings to researchers, continue to expand as new challenges in the clinical research enterprise arise. TIN resources, many available at no cost to the broader research community, are communicated via the website, webinars, and publications. US-based investigators from CTSAs who are conducting or planning to conduct a multicenter study are encouraged to submit a proposal for a TIN consultation.
